# Adverse Pregnancy Outcomes and Long-Term Risk of Chronic Diseases: Evidence from the Findings of the Tehran Lipid and Glucose Study During a Quarter of a Century

**DOI:** 10.5812/ijem-167128

**Published:** 2026-01-31

**Authors:** Marzieh Saei Ghare Naz, Maryam Farahmand, Mahsa Noroozzadeh, Mahbanoo Farhadi-Azar, Maryam Mousavi, Fereidoun Azizi, Shabahang Amirshekari, Fahimeh Ramezani Tehrani

**Affiliations:** 1 Reproductive Endocrinology Research Center, Research Institute for Endocrine Molecular Biology, Research Institute for Endocrine Sciences, Shahid Beheshti University of Medical Sciences, Tehran, Iran; 2 Endocrine Research Center, Research Institute for Endocrine Disorders, Research Institute for Endocrine Sciences, Shahid Beheshti University of Medical Sciences, Tehran, Iran; 3 Foundation for Research & Education Excellence, Vestavia Hills, AI, USA

**Keywords:** Tehran Lipid and Glucose Study, Adverse Pregnancy Outcomes, Metabolic Syndrome, Long Term Adverse Effects

## Abstract

**Context:**

Evidence supports that adverse pregnancy outcomes (APOs) might affect women's health in later life. This review summarizes the findings of the Tehran Lipid and Glucose Study (TLGS) on the association between APOs and long-term chronic diseases over the past two decades.

**Evidence Acquisition:**

This narrative review was conducted on TLGS-published articles on the association between APOs and long-term chronic disease over the last decades. The search for articles was performed on PubMed from 1999 to September 2025.

**Results:**

As of September 2025, nine peer-reviewed English-language articles have been published from the TLGS dataset. Studies showed that a history of hypertensive disorders of pregnancy (HDP) was associated with a twofold increased risk of hypertension, a threefold risk of diabetes, and a 1.3 times risk of dyslipidemia. Moreover, women with a history of preeclampsia were 3.62 times more likely to experience hypertension progression. Women with a history of gestational diabetes mellitus (GDM) had a 2.23 times risk of diabetes and a 1.85 times risk of cardiovascular disease (CVD). Having a history of preterm delivery increased the risk of chronic kidney disease (CKD) 2.68 times more than that of women without such a history. The risk of metabolic syndrome (METS) was increased by 8% in women with a history of pregnancy loss. Moreover, a history of one, two, or three APOs was associated with an increased risk of developing CVD, with the risk rising progressively as the number of APOs increased.

**Conclusions:**

Over the past two decades, the TLGS has published a number of peer-reviewed articles that collectively provide a unique perspective on the long-term health consequences of APOs. These studies indicate that pregnancy is not only an obstetric event but also a vital period for assessing women's long-term health. Women with a history of APOs require early preventive evaluations and ongoing monitoring to identify and treat chronic conditions linked to increased risks of premature mortality.

## 1. Context

Adverse pregnancy outcomes (APOs) impact about 1 in 5 live births (1). Recently, due to delayed motherhood and the increasing trend in advanced maternal age (AMA ≥ 35 years), a higher rate of APOs was expected (2). Subsequently, APOs place a greater burden on health system resources (3). Moreover, various factors are known predisposing factors for the development of APOs, including maternal behavior, parental consanguinity, and pre-existing maternal illnesses (4).

Pregnancy has been described as a physiological stress test, which may reveal hidden predispositions to disease that can remain undetected for many years (5). Evidence supports the close link between APOs and long-term maternal complications (6-8). A robust systematic review and meta-analysis demonstrated that exposure to APOs was associated with an increased risk of maternal kidney disease in the long term (9). Also, a national cohort study among more than 2 million women in Sweden with a history of at least one APO showed that these women were at increased risk of mortality (10). Pregnancy complications are also identified as one of the main determinants of long-term cardiovascular disease (CVD) outcomes (11). It is proposed that vascular, metabolic, and inflammatory complications during pregnancy can result in an elevated risk of vascular disease in the long term (12).

This study aimed to review all findings from studies conducted within the framework of the Tehran Lipid and Glucose Study (TLGS) regarding APOs and the long-term risk of chronic diseases, such as type 2 diabetes (T2DM), CVD, pre-diabetes, dyslipidemia, hypertension, metabolic syndrome (METS), and chronic kidney disease (CKD).

## 2. Evidence Acquisition

This is a narrative synthesis of TLGS publications on the association between APOs and non-communicable diseases later in life. The search was performed on PubMed databases up to September 2025. The search was performed using the search terms (Pregnancy, Complication, Pregnancy, Pregnancy Complication, Complications, Pregnancy, Adverse Birth Outcomes, Adverse Birth Outcome, Birth Outcome, Adverse, Outcome, Adverse Birth, Outcome, Pregnancy, Outcomes, Pregnancy, Pregnancy Outcomes, Diabetes Mellitus, Gestational, Gestational Diabetes Mellitus (GDM), Diabetes, Pregnancy-Induced, Diabetes Pregnancy Induced, Pregnancy-Induced Diabetes, Gestational Diabetes, Preeclampsia, Gestational Hypertension, Pregnancy Induced Hypertension, Stillbirth, Loss Early Pregnancy, Pregnancy Loss, Early, Miscarriage, Abortion, Preterm Birth, TLGS). All English-language studies investigating prospective associations and long-term outcomes in women with a history of APOs or case-control studies in the framework of the TLGS were assessed. Furthermore, studies focusing on spousal risk and other metabolic disorders in women were also included in this review.

### 2.1. Eligibility

We included all peer-reviewed original published articles in the framework of the TLGS in which: (1) At least one APO (HDP, GDM, preterm delivery, abortion/stillbirth) was reported as the exposure; and (2) at least one long-term maternal outcome (T2DM, CVD, CKD, pre-diabetes, hypertension, dyslipidemia, and METS) was reported. Two reviewers independently screened titles/abstracts and full texts for eligibility. Information on APOs was collected via self-report and then confirmed by medical documents (13). Diagnosis was based on the standard definition as follows:

• Gestational diabetes mellitus: World Health Organization 1999/national criteria based on a 75-g oral glucose tolerance test during pregnancy was used in this study.

• Hypertensive disorders of pregnancy (HDP): Preeclampsia was defined as new-onset hypertension (≥ 140/90 mmHg) after 20 weeks with proteinuria or signs of organ dysfunction, whereas gestational hypertension was defined as elevated blood pressure without proteinuria.

• Preterm delivery: Delivery before 37+0 completed weeks of gestation, based on ultrasound or last menstrual period dating.

• Pregnancy loss: A history of any abortion, miscarriage, or stillbirth was considered a pregnancy loss variable. According to the World Health Organization/International Classification of Diseases (WHO/ICD), stillbirth is the death of a fetus that has reached a birth weight of 500 g, or if birth weight is unavailable, gestational age of 22 weeks or crown-to-heel length of 25 cm (14). Abortion, according to contemporary consensus in the medical community, is the process of delivering a conceptus before the fetus's viability, which is defined as 20 weeks of pregnancy or a fetus weighing 500 grams or more (15). A miscarriage occurs spontaneously, and abortion refers to the intentional ending of pregnancy (16).

Given that all included publications were derived from the same underlying cohort, methodological quality was assessed at the cohort level rather than at the level of individual articles.

## 3. Results

**Figure 1.**
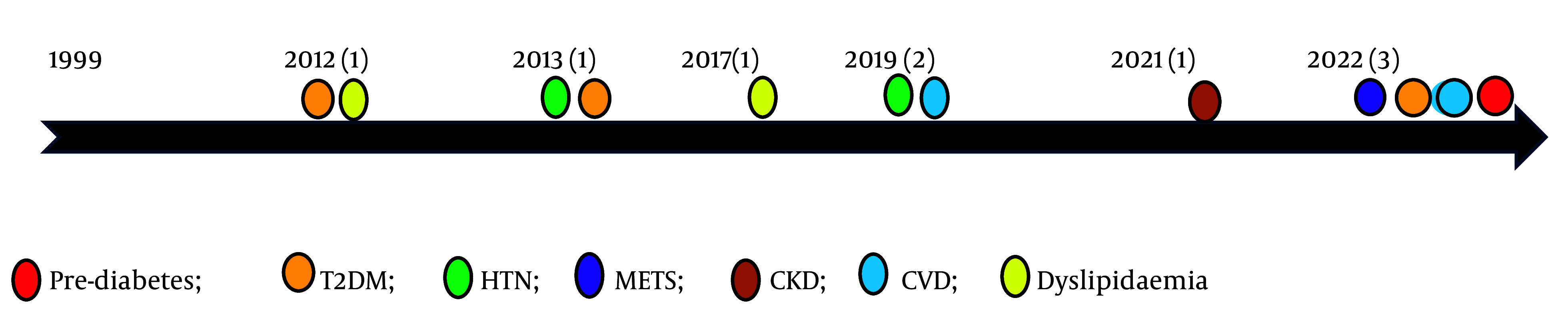
Timeline of publications by outcome

Finally, nine relevant papers were included in this review. One paper reported the association between preterm delivery and the risk of CKD, and two papers noted the association between APOs and the risk of CVD. Associations between GDM/HDP and hypertension, as well as T2DM, were described in one paper. The outcome of dyslipidemia has also been assessed in relation to GDM in two papers. The impact of preeclampsia and hypertension progression was explained in another article, while the association between pregnancy loss and pre-diabetes, diabetes, and METS was reported in a newly published paper. Figure 1 shows the timeline of publications by outcome. Table 1 shows the characteristics of included studies.

**Table 1.** Characteristics of Included Studies

Publication Year, Ref.	Exposures	Outcomes	Sample Sizes	Follow-up Duration (y)	Covariates Adjusted Factors	Effect Estimates (RR/HR/OR), 95% CIs
**2012 (** 17 **)**	Gestational diabetes	T2DM, HTN	Women with prior history of GDM = 29; women with history of macrosomia or stillbirth without GDM (MC-ST) = 570; age- and BMI-matched controls = 628	9	Concentration of metabolic factors at the initiation of the study	27.3% of women with GDM and 9.5% among the control group had T2DM; 7.4% of the MC-ST group and 8.9% of control group had T2DM during; the incidence of HTN or dyslipidemia were not significantly different between groups.
**2013 (** 18 **)**	Hypertensive pregnancy disorders	Hypertension, T2DM mellitus and dyslipidemia	Case = 226 control = 226	10	Basic cardiovascular and metabolic characteristics	Women with a history of HPD, compared with control, had a threefold increased risk for T2DM (95% CI:1.8 - 5.2), a twofold increased risk for hypertension (95% confidence interval [CI]: 1.4 - 3.2), and a 1.3-fold increased risk for dyslipidemia (95% CI: 1.2 - 1.5).
**2017 (** 19 **)**	Gestational diabetes	Trend of lipid parameters changes	GDM = 289; women without GDM = 1183	15	Age, BMI, smoking	Person-time dyslipidemia incidence rate in women with previous GDM was 0.067 (CI: 0.038, 0.096) with a median progression time of 2.13 years and for those without GDM was 0.059 (CI: 0.046, 0.072) with the median time of 2.31 years (P = 0.214)
**2019 (** 20 **)**	Gestational diabetes	CVD	2547 cases	14.1	Age, body mass index, smoking (for men), maternal, parity, miscarriage, physical activity, hypertension and hypercholesterolemia, and diabetes mellitus	History of GDM was associated with adjusted hazard ratio (HR), 95% CI of 1.85 (1.38 - 2.48) and 1.29 (0.96 - 1.75) for CVD in models 1 and 2, respectively.
**2019 (** 21 **)**	Preeclampsia	Blood pressure	3022 eligible women;	15	Age, BMI, TG, and HDL	The risk of HTN progression in women with a history of PE was higher (HR: 3.62; 95% CI: 2.70 - 4.62) compared to women in non-PE group.
**2021 (** 22 **)**	Preterm delivery	CKD	Women with a history of at least one preterm delivery = 212; women with term delivery = 2823	16	Smoking, parity, age at first delivery, BMI, educational level, preeclampsia, and GDM	Women with a history of preterm delivery were at increased risk of CKD (HR: 2.62; 95% CI 1.02, 7.05).
**2022 (** 23 **)**	APOs	CVD	4013 women	19	Age at baseline, antihypertensive use, Serum TC, high-density lipoprotein cholesterol and current smoking, T2DM,	CVD event in women with a history of multiple APOs compared with cases with only 1 APO (1 APO: hazard ratio [HR] = 1.22; 2 APOs: HR; 1.94; ≥ 3 APOs: HR = 2.48).
**2022 (** 24 **)**	Pregnancy loss	Prediabetes, diabetes and METS	2765 couples	15	Age, WHtR, BMI, education, parity, number of pregnancy loss, SBP, FBS, TG, TC, LDL, and family history of diabetes.	Females with history of pregnancy loss were at an increased the risk of METs [RR = 1.08; 95%CI: (1.02, 1.14)] than females without such a history.
**2022 (** 25 **)**	GDM and hypertensive disorder of pregnancy	Diabetes and hypertension	3650 pairs of spouses	12 - 13	Age, waist-to-height ratio, physical activity, smoking, and parity	Having histories of both GDM and HDP result in increased risk of females to 3.05 (95 % CI: 1.43, 6.52) times of their spouses for diabetes. Also, females with history of GDM (HR: 3.51, 95 % CI: 2.23, 5.53), or HDP (HR: 2.80, 95 % CI: 1.72, 4.56) were at higher risk of T2DM compared with females who never had GDM or HDP.

Abbreviations: PE, preeclampsia; T2DM, type 2 diabetes; HTN, hypertension; GDM, gestational diabetes mellitus; HDP, hypertensive disorders of pregnancy; CVD, cardiovascular disease; METS, metabolic syndrome; CKD, chronic kidney disease; APOs; adverse pregnancy outcomes; BMI, Body Mass Index.

According to the Newcastle-Ottawa Quality Assessment Form for Cohort Studies: (1) The population of exposed and non-exposed groups was representative. Ascertainment of exposure was based on the standard protocol of the TLGS; (2) analysis controlled for confounders; (3) outcome ascertainment, completeness of follow-up, and sample size and statistical power had good quality.

### 3.1. Hypertensive Disorders of Pregnancy

Hypertensive disorders of pregnancy are one of the most frequent complications of pregnancy and the leading cause of maternal mortality worldwide (26). One publication of TLGS on the association of APO with metabolic disorders in 2013 was a case-control study on 226 women with a history of HDP as the case group, and 226 age- and body-mass-index-matched women with no history of HDP. The result of this study showed that having a history of HDP was associated with increased risk of hypertension (relative risk: 2.12; 95% confidence interval [CI]: 1.4 - 3.2), T2DM (3.1; 95% CI: 1.8 - 5.2), and dyslipidemia (1.3; 95% CI: 1.2 - 1.5) (18). In 2019, another study using TLGS data on 355 women with a history of preeclampsia (PE) and 2667 non-PE women was published. This study demonstrated that women with a history of PE were at increased risk of hypertension progression (hazard ratio: 3.62; 95% CI: 2.70 - 4.62; P < 0.001) (21). Another publication in 2022 among 2820 females and their spouses showed that in women with histories of both GDM and HDP, the risk of hypertension increases to 3.05 (95% CI: 1.43, 6.52) times that of their spouses (25).

### 3.2. Gestational Diabetes Mellitus

Gestational diabetes mellitus is a common APO that contributes to the development of other pregnancy complications (27). The publications on GDM and maternal health later in life were among the most common studies on pregnancy outcomes. The first publication in 2012 using data of TLGS among 29 women with prior history of GDM, 570 women with history of macrosomia or stillbirth without GDM (MC-ST), and 628 age- and BMI-matched controls showed that T2DM was diagnosed in 27.3% of women with GDM, 7.4% of the MC-ST group, and 9.5% of the control group (P < 0.05). The incidence of hypertension and dyslipidemia were not significantly different between groups (17). Five years later, Minooee et al. reported that the person-time incidence rate of dyslipidemia among women with a history of GDM was 0.067 (19). Moreover, a study among 2820 females and their spouses demonstrated that women with a history of GDM (hazard ratio: 3.51, 95% CI: 2.23 - 5.53) were at a higher risk of developing diabetes compared to females who had never had GDM or HDP (25). As mentioned above, this exposure in combination with HDP was associated with increased risk of hypertension later in life. A study also on 4308 women and their spouses showed that women with a history of GDM had a higher risk of CVD [hazard ratio, 95% CI: 1.85 (1.38 - 2.48)] (20).

### 3.3. Preterm Delivery

Preterm birth is not only a leading cause of neonatal and infant morbidity and mortality but also threatens maternal future health (28, 29). There is only one publication on the association between preterm delivery and chronic disease risk later in life, using data from the TLGS. The study included 212 women with a history of at least one preterm delivery and 2823 women with term deliveries. The results showed that the risk of CKD among women with a history of at least one preterm delivery was higher than that of women without this history (hazard ratio: 2.68; 95% CI: 1.02, 7.05; P = 0.04) (22).

### 3.4. Pregnancy Loss

It is estimated that 44 pregnancy losses occur each minute (30). A publication with exposure to pregnancy loss (history of any abortion or miscarriage, or stillbirth) and metabolic disorders as outcomes on the TLGS dataset was found. This study among 2765 couples with and without a history of pregnancy loss showed that women with a history of pregnancy loss experienced more pre-diabetes (50% vs. 45.5%), diabetes (28.9% vs. 21.3%), and METS (70% vs. 60.1%) than women without such a history. Females with a history of pregnancy loss experienced more pre-diabetes (50% vs. 45.5%), diabetes (28.9% vs. 21.3%), and METS (70% vs. 60.1%) than females without such a history. Females with a history of pregnancy loss were at an elevated risk of METS than females without a history of pregnancy loss (relative risk = 1.08; 95% CI: 1.02, 1.14; P = 0.01) (24).

### 3.5. Adverse Pregnancy Outcomes

**Figure 2.**
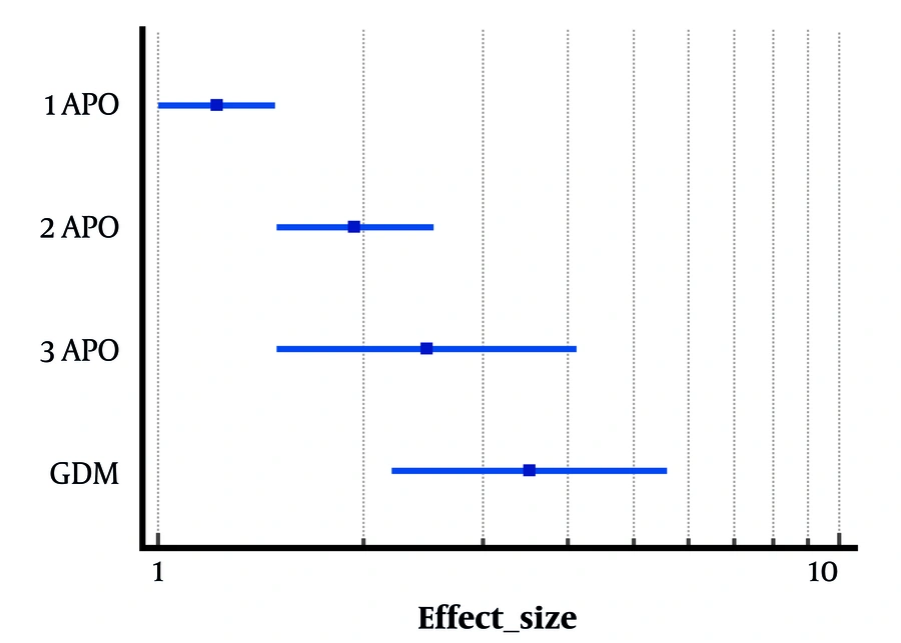
Risk cardiovascular disease (CVD) according to the exposure with adverse pregnancy outcome among participants of Tehran Lipid and Glucose Study (TLGS)

A publication in the Journal of the American Heart Association in 2022, using the TLGS dataset, showed that among 4013 women, 1484 (36.98%) reported one APO, while 395 (9.84%) reported multiple APOs. The results of that study also showed that a history of multiple APOs, compared with cases with only one APO, was associated with a greater risk of CVD (one APO: Hazard ratio = 1.22; two APOs: Hazard ratio = 1.94; ≥3 APOs: Hazard ratio = 2.48) (23). This robust study, which shows the added value of APOs on the Framingham risk score, was cited in the 2024 European Society of Cardiology (ESC) Guidelines for the management of elevated blood pressure and hypertension (31) as well as in the Journal of the American Heart Association (JAHA) spotlight on pregnancy and its impact on maternal and offspring cardiovascular health (32). Figure 2 shows the risk of CVD according to the exposure to APOs among participants of TLGS.

## 4. Conclusions

Over the past two decades, the TLGS has published a number of peer-reviewed articles that provide a unique perspective on the long-term health consequences of APOs. These studies indicate that pregnancy is not only an obstetric event but also a vital period for assessing women's long-term health. Clinicians should consider special care attention, including early chronic disease risk assessment, lifestyle counseling, and cardiometabolic condition monitoring for women with a history of APOs, acknowledging that future studies should guide specific interventions. Further research is needed to clarify underlying mechanisms, differentiate the impact of specific pregnancy complications, and evaluate preventive strategies, thereby informing more precise, evidence-based clinical recommendations.

## References

[1] Freaney PM, Harrington K, Molsberry R, Perak AM, Wang MC, Grobman W (2022). Temporal Trends in Adverse Pregnancy Outcomes in Birthing Individuals Aged 15 to 44 Years in the United States, 2007 to 2019.. J Am Heart Assoc..

[2] Li H, Fan C, Mubarik S, Nabi G, Ping YX, Nawsherwan (2022). The trend in delayed childbearing and its potential consequences on pregnancy outcomes: a single center 9-years retrospective cohort study in Hubei, China.. BMC Pregnancy Childbirth..

[3] Law A, McCoy M, Lynen R, Curkendall SM, Gatwood J, Juneau PL (2015). The prevalence of complications and healthcare costs during pregnancy.. J Med Econ..

[4] Doke PP, Palkar SH, Gothankar JS, Patil AV, Chutke AP, Pore PD (2021). Association between adverse pregnancy outcomes and preceding risk factors: a cross-sectional study from Nashik District, India.. BMC Pregnancy Childbirth..

[5] McNestry C, Killeen SL, Crowley RK, McAuliffe FM (2023). Pregnancy complications and later life women's health.. Acta Obstet Gynecol Scand..

[6] Torosyan N, Aziz D, Quesada O (2022). Long-term sequelae of adverse pregnancy outcomes.. Maturitas..

[7] Crump C, Sundquist J, Sundquist K (2025). Adverse Pregnancy Outcomes and Long-Term Risk of Heart Failure in Women: National Cohort and Co-Sibling Study.. JACC Heart Fail..

[8] Bodunde EO, Buckley D, O'Neill E, Al Khalaf S, Maher GM, O'Connor K (2025). Pregnancy and birth complications and long-term maternal mental health outcomes: A systematic review and meta-analysis.. BJOG..

[9] Barrett PM, McCarthy FP, Kublickiene K, Cormican S, Judge C, Evans M (2020). Adverse Pregnancy Outcomes and Long-term Maternal Kidney Disease: A Systematic Review and Meta-analysis.. JAMA Netw Open..

[10] Crump C, Sundquist J, Sundquist K (2024). Adverse Pregnancy Outcomes and Long-Term Mortality in Women.. JAMA Intern Med..

[11] Rabadia SV, Heimberger S, Cameron NA, Shahandeh N (2025). Pregnancy Complications and Long-Term Atherosclerotic Cardiovascular Disease Risk.. Curr Atheroscler Rep..

[12] Neiger R (2017). Long-Term Effects of Pregnancy Complications on Maternal Health: A Review.. J Clin Med..

[13] Ramezani Tehrani F, Behboudi-Gandevani S, Rostami Dovom M, Farahmand M, Minooee S, Noroozzadeh M (2018). Reproductive Assessment: Findings from 20 Years of the Tehran Lipid and Glucose Study.. International Journal of Endocrinology and Metabolism..

[14] Tavares Da Silva F, Gonik B, McMillan M, Keech C, Dellicour S, Bhange S (2016). Stillbirth: Case definition and guidelines for data collection, analysis, and presentation of maternal immunization safety data.. Vaccine..

[15] Shakhatreh HJM, Salih AJ, Aldrou K, Alazzam FAF, Issa MSB (2022). Medico-Legal Aspects of Abortion: Updates of the Literature.. Med Arch..

[16] Wang Y, Tang Y, Wang G, Wei R, Liu L, Lu C (2025). Epidemiological trends in abortion and miscarriage between 1990 and 2019.. Reprod Health..

[17] Tehrani FR, Hashemi S, Hasheminia M, Azizi F (2012). Follow-up of women with gestational diabetes in the Tehran Lipid and Glucose Study (TLGS): a population-based cohort study.. J Obstet Gynaecol Res..

[18] Hashemi S, Ramezani Tehrani F, Mehrabi Y, Azizi F (2013). Hypertensive pregnancy disorders as a risk factor for future cardiovascular and metabolic disorders (Tehran Lipid and Glucose Study).. J Obstet Gynaecol Res..

[19] Minooee S, Ramezani Tehrani F, Rahmati M, Mansournia MA, Azizi F (2017). Dyslipidemia incidence and the trend of lipid parameters changes in women with history of gestational diabetes: a 15-year follow-up study.. Endocrine..

[20] Kabootari M, Hasheminia M, Guity K, Ramezankhani A, Azizi F, Hadaegh F (2019). Gestational diabetes mellitus in mothers and long term cardiovascular disease in both parents: Results of over a decade follow-up of the Iranian population.. Atherosclerosis..

[21] Amiri M, Ramezani Tehrani F, Rahmati M, Behboudi-Gandevani S, Azizi F (2019). Changes over-time in blood pressure of women with preeclampsia compared to those with normotensive pregnancies: A 15 year population-based cohort study.. Pregnancy Hypertens..

[22] Saei Ghare Naz M, Rahmati M, Azizi F, Ramezani Tehrani F (2021). Risk of chronic kidney disease in women with a history of preterm delivery: Tehran Lipid and Glucose Study.. J Nephrol..

[23] Saei Ghare Naz M, Sheidaei A, Aflatounian A, Azizi F, Ramezani Tehrani F (2022). Does Adding Adverse Pregnancy Outcomes Improve the Framingham Cardiovascular Risk Score in Women? Data from the Tehran Lipid and Glucose Study.. J Am Heart Assoc..

[24] Rahmati M, Saei Ghare Naz M, Azizi F, Ramezani Tehrani F (2022). Pregnancy loss and subsequent risk of prediabetes, diabetes and metabolic syndrome in couples: Tehran lipid and glucose study.. J Transl Med..

[25] Saei Ghare Naz M, Sheidaei A, Azizi F, Ramezani Tehrani F (2022). Gestational diabetes mellitus and hypertensive disorder of pregnancy play as spouse-pair risk factors of diabetes and hypertension: Insights from Tehran Lipid and Glucose Study.. J Diabetes Complications..

[26] Beaulac A, Cumyn A, Côté A, Caron N (2025). Hypertensive disorders of pregnancy: A narrative review.. Canadian Journal of General Internal Medicine..

[27] Ye W, Luo C, Huang J, Li C, Liu Z, Liu F (2022). Gestational diabetes mellitus and adverse pregnancy outcomes: systematic review and meta-analysis.. BMJ..

[28] Mitrogiannis I, Evangelou E, Efthymiou A, Kanavos T, Birbas E, Makrydimas G (2023). Risk factors for preterm birth: an umbrella review of meta-analyses of observational studies.. BMC Med..

[29] Wu P, Gulati M, Kwok CS, Wong CW, Narain A, O'Brien S (2018). Preterm Delivery and Future Risk of Maternal Cardiovascular Disease: A Systematic Review and Meta-Analysis.. J Am Heart Assoc..

[30] Quenby S, Gallos ID, Dhillon-Smith RK, Podesek M, Stephenson MD, Fisher J (2021). Miscarriage matters: the epidemiological, physical, psychological, and economic costs of early pregnancy loss.. Lancet..

[31] McEvoy JW, McCarthy CP, Bruno RM, Brouwers S, Canavan MD, Ceconi C (2024). 2024 ESC Guidelines for the management of elevated blood pressure and hypertension.. Eur Heart J..

[32] Russell MW (2022). JAHA Spotlight on Pregnancy and Its Impact on Maternal and Offspring Cardiovascular Health.. J Am Heart Assoc..

